# Role of *HIF-1α-miR30a-Snai1* Axis in Neonatal Hyperoxic Lung Injury

**DOI:** 10.1155/2019/8327486

**Published:** 2019-10-22

**Authors:** Yuhao Zhang, Xiaoyu Dong, Krithika Lingappan

**Affiliations:** Department of Pediatrics, Section of Neonatology, Texas Children's Hospital, Baylor College of Medicine, Houston, Texas, USA

## Abstract

Bronchopulmonary dysplasia (BPD) is characterized by a severe impairment in lung alveolarization and vascular development. We have previously shown that pulmonary angiogenesis is preserved in hyperoxia-exposed female mice accompanied by increased *miR-30a* expression, which is a proangiogenic miRNA. Also, *miR-30a* expression is decreased in human BPD. HIF-1*α* plays an essential role in postnatal lung development, especially in recovery from hyperoxic injury. *Snai1* activation promotes pathological fibrosis through many mechanisms including Endo-MT, which may in turn adversely impact lung vascular development. Our objective was to test the hypothesis that higher miR-30a expression through *HIF-1α* decreases *Snai1* expression in females and attenuates injury in the developing lung. Neonatal male and female mice (C57BL/6) were exposed to hyperoxia (P1-5, 0.95 FiO_2_) and euthanized on P21. Neonatal human pulmonary microvascular endothelial cells (HPMECs; 18-24-week gestation donors; 3/group either sex) were subjected to hyperoxia (95% O_2_ and 5% CO_2_) or normoxia (air and 5% CO_2_) up to 72 h. *Snai1* expression was measured in HPMECs *in vitro* and in neonatal mouse lungs *in vivo*. Also, *Snai1* expression was measured in HPMECs after *miR-30a* mimic and *miR-30a* inhibitor treatment. To further establish the potential regulation of *miR-30a* by *Hif-1α*, *miR-30a* expression after Hif-1*α* inhibition was measured in HPMECs. *In vivo*, *Snai1* expression was decreased in neonatal female lungs compared to males at P7. Increased *Snai1* expression was seen in male HPMECs upon exposure to hyperoxia *in vitro*. Treatment with the *miR-30a* mimic decreased *Snai1* expression in HPMECs, while *miR-30a inhibition* significantly increased *Snai1* expression in HPMECs. siRNA-mediated loss of *Hif-1α* expression in HPMECs decreased *miR-30a expression*. *Hif-1α* may lead to differential sex-specific miR-30a expression and may contribute to protection from hyperoxic lung injury in female neonatal mice through decreased *Snai1* expression.

## 1. Introduction

With the increasing survival of extremely premature babies, the incidence of bronchopulmonary dysplasia (BPD) has remained steady, despite the advances in neonatal intensive care. BPD is the cause of significant morbidity and leads to prolonged impairment in lung function in this group of patients. Many prenatal and postnatal factors are involved in the pathogenesis of BPD, and exposure to supraphysiological concentrations of oxygen contributes to its development. Murine models have utilized varying durations of postnatal hyperoxia exposure to simulate the human disease in neonatal mice [1, 2].

Bronchopulmonary dysplasia (BPD) is characterized mainly by an arrest in lung development with severe impairment of alveolar septation and vascular development, and pathological fibrosis in severe cases [3–6]. Histopathological analysis in neonates with “new BPD” shows evidence of variable interstitial fibroproliferation compared to extensive fibroproliferation in “old BPD” [7]. BPD disproportionately affects male infants compared to females. The molecular mechanisms underlying this sex bias are not known. We have previously shown that alveolarization and pulmonary angiogenesis is preserved in hyperoxia-exposed female neonatal mice compared to males [8]. The protective effect in females is accompanied by an increase in pulmonary *miR-30a* expression in females [9]. *miR-30a* has proangiogenic, anti-inflammatory, and antifibrotic effects in many diseases processes [10–17], including those affecting the lung. Significantly, *miR-30a* expression is decreased in human patients with BPD [9]. The upstream regulation of *miR-30a* leading to differential expression in males and females has not been elucidated. Previously published work has shown that *Hif-1α* may increase *miR-30a* expression [18]. HIF-1*α* plays a vital role in postnatal lung development [19], especially in recovery from hyperoxic injury [20] and acute lung injury [21]. Overexpression of *Hif-1α* in hyperoxia-exposed neonatal mice attenuates lung injury [20]. *HIF-1α* binding to its target genes is higher in female lungs after hyperoxia exposure [22]. Whether *HIF-1α* increases *miR-30a* expression in the pulmonary microvascular endothelium is not known. *Snai1* is a transcriptional repressor and a profibrotic molecule [23–28]. Studies have shown that *miR-30a* downregulates *Snai1* [10, 14, 17].

However, the mechanistic role of the intersection between HIF-1*α*, miR-30a, and Snai1 in neonatal hyperoxic lung injury has not been studied. Our objective was to test the hypothesis that HIF-1*α* increases the miR-30a expression in females and decreases the Snai1 expression in the developing lung.

## 2. Methods

### 2.1. Animals

All experiments were performed per relevant guidelines and regulations. The care of animals was as per the 8^th^ edition of the guide for the care and use of laboratory animals and other IACUC protocols. Timed pregnant C57BL/6J WT mice were obtained from Charles River Laboratories (Wilmington). The sex in neonatal mouse pups was determined as described before and with PCR analysis for the Sry gene [8].

#### 2.1.1. Mouse Model of BPD

Mouse pups were exposed to normoxia (21% O_2_) or hyperoxia (95% O_2_), within 12 h of birth for five days. Neonatal mice are at the saccular stage of lung development during this period, equivalent to 26-36 weeks in human gestation. The dams were rotated between air- and hyperoxia-exposed litters every 24 hours to prevent oxygen toxicity in the dams. Oxygen exposure was conducted in plexiglass chambers as previously described [8]. Mice were euthanized on P7 and P21 (after recovery in room air). The mice were euthanized with sodium pentobarbital, 100 mg/kg, i.p. For the *in vivo* experiments, neonatal mice from different litters were randomly allocated to room air or hyperoxia. The experimental unit was a single neonatal mouse pup and data from 4-6 animals/group were used for analysis.

#### 2.1.2. Cell Culture and Hyperoxia Treatment

Neonatal human pulmonary microvascular endothelial cells (HPMEC; male: #10885, #11367, and #10899; female: #5016, #10160, and #10169; 6 biological replicates in total) were purchased from ScienCell and maintained in Endothelial Cell Medium (Cat # 1001, ScienCell) at 37°C in 5% CO_2_. The gestational age of the donors varied from 18 to 24 weeks. Male and female HPMECs were used from passages 3 to 5 to ensure their endothelial characteristics. Male or female HPMEC cells (1 × 10^5^) were seeded in a 6 mm dish. 24 h later, these cells were incubated at 37°C in room air condition (21% O_2_, 5% CO_2_) or in hyperoxia (95% O_2_, 5% CO_2_) as described before for 72 h. For measuring SNAI1 expression, both male and female HPMECs were used. For the rest of the *in vitro* experiments, female HPMECs were used as the miR-30a expression was increased only in female HPMECs [9].

#### 2.1.3. miRNA and Plasmid Transfection in HPMECs

miR30a-5p mimic (HMI0454), miR30a-5p inhibitor (HSTUD0454), Hif-1*α*-siRNA (3017635209), and negative control siRNA (NCSTUD001) were purchased from MilliporeSigma. Hif-1*α* overexpression plasmid (#18948) was purchased from Addgene. pcDNA3.1 (V79020) plasmid was purchased from Thermo Fisher. MicroRNA negative control (30 pmol), miR30a-5p mimic (30 pmol), or miR30a-5p inhibitor (30 pmol) were used for HPMEC transfection separately using a Lipofectamine 2000 reagent (Cat #11668019; 6 *μ*l per well of a 6-well plate). Hif-1*α* plasmid (2 *μ*g) with or without miR30a-5p inhibitor (60 pmol) was transfected using a Lipofectamine 2000 reagent (6 *μ*l per well of a 6-well plate). pcDNA3.1 plasmid (2 *μ*g/well) was transfected as a negative control of Hif-1*α*. After six hours, fresh medium was added to the plate. After 48 h incubation in room air (21% O_2_, 5% CO_2_), cells were harvested for later use.

#### 2.1.4. Quantitative PCR

Total RNA and microRNA was extracted from the lung tissues and cell lines using TRIzol (Cat #15596026, Thermo Fisher) and chloroform (Cat #C2432, Millipore-Sigma) and then treated with DNase I (Cat #K1622, Invitrogen). cDNA was prepared using a RevertAid Reverse Transcriptase (Thermo Fisher). MicroRNA cDNA was generated using a MystiCq microRNA cDNA Synthesis Mix (MilliporeSigma). Quantitative PCR was performed using the QuantStudio 7 Flex Real-Time PCR Detection System (Thermo Fisher) and SYBR Green (Cat #1725274, Bio-Rad). The thermal cycling conditions used were as follows: one cycle at 95°C for 1 min, 40 cycles at 95°C for 15 s, and one cycle at 60°C for 30 s. The primers used in the real-time PCR test were listed as follows: Snai1 (NM_005985, human) forward primer: GCGAGCTGCAGGACTCTAAT, reverse primer: GGACAGAGTCCCAGATGAGC; Snai1 (NM_011427, mice) forward primer: CACCCTCATCTGGGACTCTC, reverse primer: GAGCTTTTGCCACTGTCCTC; Hif-1*α* (NM_001243084, human) forward primer: GATGTAATGCTCCCCTCACC, reverse primer: CTTGATTGAGTGCAGGGTCA; Hif-1*α* (NM_001313919, mice) forward primer: ATTCTCCAAGCCCTCCAAGT, reverse primer: TCATCAGTGGTGGCAGTTGT; *β*-actin (NM_001101, human) forward primer: CATCGAGCACGGCATCGTCA, reverse primer: TAGCACAGCCTGGATAGCAAC; *β*-actin (NM_007393, mice) forward primer: GATCTGGCACCACACCTTCT, reverse primer: GGGGTGTTGAAGGTCTCAAA; beta 2-microglobulin (NM_009735.3, mouse) forward primer: CTGACCGGCCTGTATGCTAT, reverse primer: CCGTTCTTCAGCATTTGGAT; and beta 2-microglobulin (NM_00408.3, human) forward primer: TGCTGTCTCCATGTTTGATGTATCT, reverse primer: TCTCTGCTCCCCACCTCTAAGT. miR30a-5p (MIRAP00079), MystiCq Universal PCR (MIRUP), and U6 (MIRCP00001) primers were purchased from MilliporeSigma. Relative mRNA levels were calculated using the 2^-*ΔΔ*CT^ method and normalized by *β*-actin or beta 2-microglobulin in the same sample.

#### 2.1.5. Western Immunoblotting

Protein was isolated from murine lung tissue and HPMECs using RIPA buffer (Thermo Fisher) containing protease mixture inhibitors (Thermo Fisher). Proteins were separated by 4–12% sodium dodecyl sulfate polyacrylamide gel electrophoresis, then transferred to a PVDF membrane using a Mini-PROTEAN Tetra Cell system (Bio-Rad). The following primary antibodies were used: goat anti-Snai1 (1 : 1000, Abcam, ab53519), rabbit anti-*β*-actin (1 : 5000, Cell Signaling, 4970), and rabbit anti-vinculin (1 : 1000, Cat#4650, Cell Signaling). Pierce ECL plus a Western blotting substrate (Cat #32132, Thermo Fisher) was used for visualizing immunoreactive protein bands. The protein bands were normalized by *β*-actin or vinculin on the same membrane.

#### 2.1.6. Statistical Analysis

GraphPad version 7 was used for the analysis of our data. Data are expressed as means ± SD. Data were analyzed by two-way ANOVA to test for the independent effects of sex and hyperoxia and to look for any interaction (sex × hyperoxia) or by Mann-Whitney *U* test. Multiple-comparison testing (Bonferroni) was performed if statistical significance (*P* < 0.05) was noted by ANOVA.

## 3. Results

### 3.1. Differential Sex-Specific Expression of Snai1 in Neonatal Mice after Postnatal Hyperoxia Exposure

**Figure 1:**
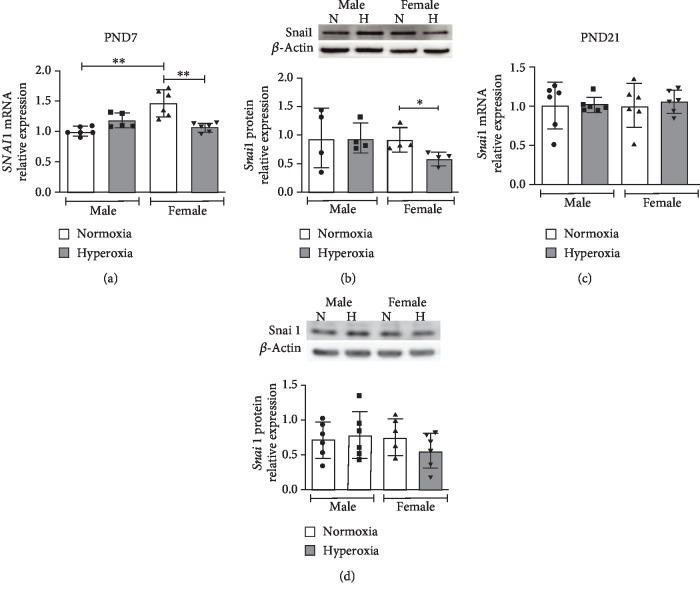
Differential sex-specific expression of *Snai1* in neonatal mice after postnatal hyperoxia exposure: *Snai1* mRNA (*β*-actin as housekeeping gene) and protein expression neonatal male and female mice exposed to hyperoxia (P1-5, 0.95 FiO_2_) during the saccular stage of lung development. *Snai1* mRNA (a, c) and protein expression (b, d) were measured in the lungs at P7 (a, b) immediately after hyperoxia exposure and at P21 (c, d) after recovery in normoxia. Values are means ± SD. *n* = 5 − 6 mice per group. Significant differences between groups indicated by ^∗^*P* < 0.05 and ^∗∗^*P* < 0.01.

WT male and female neonatal mice were exposed to hyperoxia (0.95 FiO_2_ from P1-5) during the saccular stage of lung development, and Snai1 mRNA and protein expression were measured at P7 (early) and P21 (during recovery in normoxia) and compared to respective normoxic controls. *Snai1 mRNA* expression (*β*-actin as housekeeping gene) was decreased in female neonatal mice at P7 after hyperoxia exposure compared to female normoxic controls. No difference in expression was seen in males (Figure 1(a)). The results were similar with another housekeeping gene (*β*2-microglobulin) as shown in Supp. [Supplementary-material supplementary-material-1]. Similarly, Snai1 protein expression in the lungs was also decreased in females compared to female normoxic controls and compared to hyperoxia-exposed male mice (Figure 1(b)). Snai1 mRNA and protein expression did not show statistically different expression levels between any of the groups at P21 (Figures 1(c) and 1(d)). Protein expression was repeated with vinculin as the loading control; female mice tended to have lower Snai1 protein in the lungs at P7 compared to males, and in HPMECs, males tended to have a greater increase in Snai1 expression. These results however, were not statistically significant (Supp. [Supplementary-material supplementary-material-1]).

### 3.2. Snai1 mRNA and Protein Expression Are Increased in Male Human Pulmonary Microvascular Endothelial Cells upon Exposure to Hyperoxia

**Figure 2:**
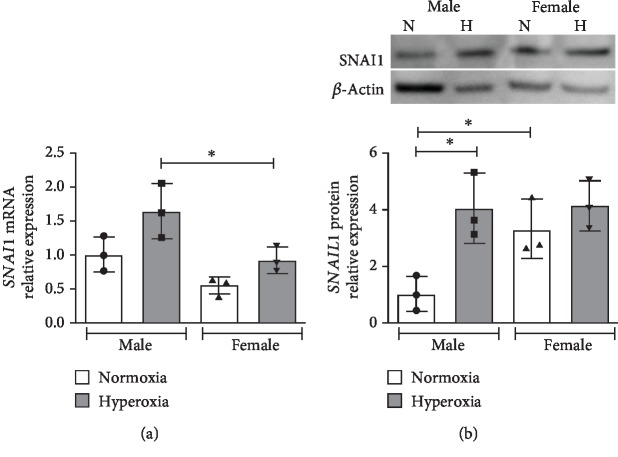
*Snai1* mRNA and protein expression is increased in human pulmonary microvascular endothelial cells upon exposure to hyperoxia: *SNAI1* mRNA (a) and protein expression (b) were measured in neonatal human pulmonary microvascular endothelial cells exposed to normoxia or hyperoxia (0.95 FiO_2_ up to 72 hrs). Values are mean ± SD. *n* = 3 per group. Significant differences between indicated groups are indicated by ^∗^*P* < 0.05.

Next, we wanted to elucidate the response to hyperoxia in neonatal human pulmonary microvascular endothelial cells (HPMECs). These cells were exposed to hyperoxia (0.95 FiO_2_) for up to 72 hours, and SNAI1 mRNA (Figure 2(a)) and protein expression (Figure 2(b)) were measured. *SNAI1* mRNA expression was significantly higher in male HPMECs compared to female. Male HPMECs showed increased SNAIL1 protein upon exposure to hyperoxia, while female HPMECs did not show this change.

### 3.3. Snai1 Regulation by miR-30a

**Figure 3:**
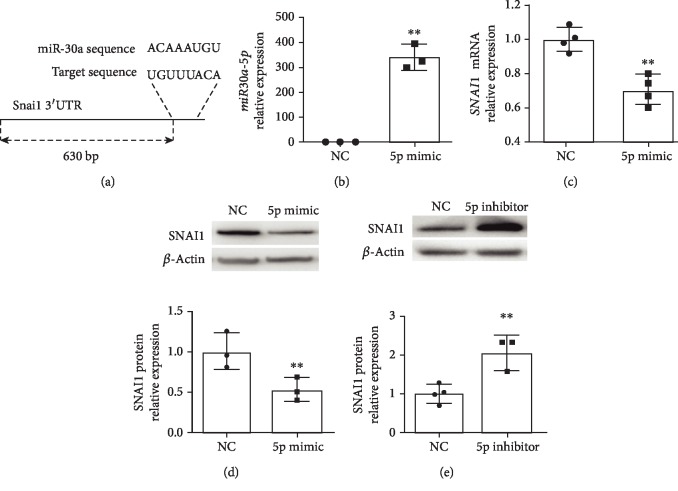
*miR30a-5p* decreases SNAI1 expression in female neonatal human pulmonary microvascular endothelial cells: (a) TargetScan reported *miR-30a* binding sites 630-637 bp in the 3′UTR of SNAI1. Treatment with the miR30a-5p mimic increased the miR30a-5p expression (b) in HPMECs. *SNAI1* mRNA (c) and protein (d) expressions were decreased in neonatal HPMECs after *miR30a-5p* overexpression. In contrast, treatment with the miR30a-5p inhibitor increased SNAI1 protein (e) expression (*n* = 3 − 4 experimental replicates/group). Values are means ± SD. Significant differences between nontreated controls are indicated by ^∗^*P* < 0.05 and ^∗∗^*P* < 0.01.

TargetScan reported *miR-30a* binding sites 630-637 bp in the 3′UTR of SNAI1 (Figure 3(a)). *miR30a-5p* expression was increased in female HPMECs using the *miR30a-5p* mimic (Figure 3(b)). Following treatment with the mimic, miR30-5p expression was increased in female HPMECs. *SNAI1* mRNA (Figure 3(c)) and protein expression (Figure 3(d)) were decreased following miR30a-5p overexpression. In contrast, SNAI1 protein expression was increased following treatment with a miR30a-5p inhibitor (Figure 3(e)).

#### 3.3.1. Regulation of miR30a-5p by HIF-1*α*

**Figure 4:**
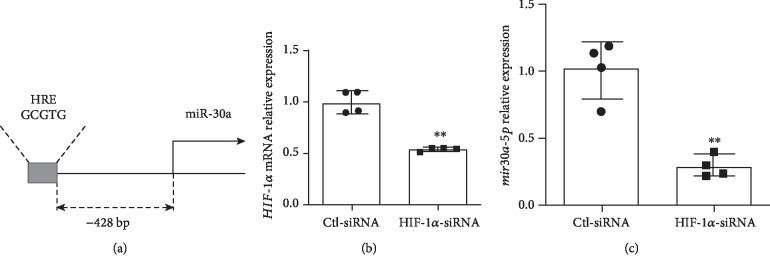
*miR-30a* regulation by HIF-1*α*. (a) Motif search revealed a hypoxia response element [AG]CGTG site 428 base pairs upstream of *miR-30a*, based on the mm10 genome build. (b) *HIF-1α* mRNA expression in female HPMECs after *HIF-1α* knockdown using siRNA (Ctl-siRNA: control siRNA). (c) *miR30a-5p* expression in female HPMECs after *HIF-1α* knockdown. Values are expressed as mean ± SD. *N* = 4 experimental replicates for each group in each test. Significant differences from baseline are indicated by ^∗∗^*P* < 0.01.

We have previously shown that *Hif-1α* expression was increased in female mice at P7 and decreased in male mice at P21 after hyperoxia exposure [29] and that *HIF-1α* binding to its target genes is more significant in female lungs after hyperoxia exposure [22]. HIF-1*α* protein expression was decreased in both male and female HUVECs upon exposure to hyperoxia [29]. Motif analysis revealed a hypoxia response element (HRE) [AG]CGTG site 428 base pairs upstream of *miR-30a* (Figure 4(a)). To further establish the potential regulation of *miR-30a* by Hif-1*α*, we used the siRNA-mediated loss of the *Hif-1α* expression in female human pulmonary microvascular endothelial cells and its effect on miR30a-5p expression. Treatment of female HPMECs with *HIF-1α* siRNA significantly decreased *HIF-1α* expression (Figure 4(b)). Also, the expression of miR30a-5p was decreased in pulmonary microvascular endothelial cells transfected with *HIF-1α* siRNA compared to cells treated with scrambled siRNA (Figure 4(c)).

#### 3.3.2. SNAI1 Expression following HIF-1*α* Silencing in Female Human Pulmonary Microvascular Endothelial Cells

**Figure 5:**
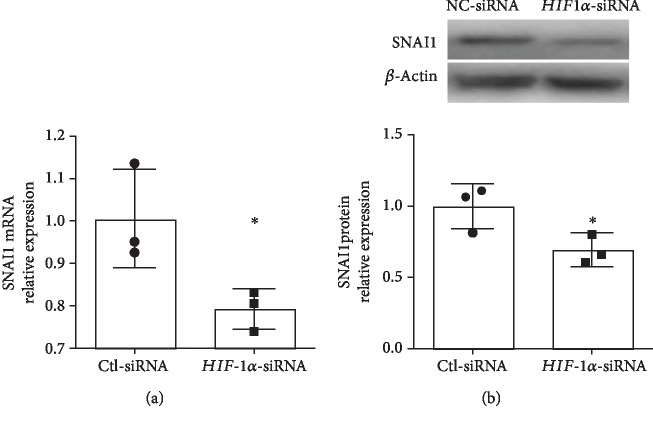
*HIF-1α* knockdown decreased *SNAI1* mRNA and protein expression in neonatal female human pulmonary microvascular endothelial cells. *SNAI1* mRNA (a) and protein (b) expression in HPMECs after *HIF-1α* knockdown using siRNA (Ctl-siRNA: control siRNA). Values are expressed as mean ± SD. *N* = 3 experimental replicates for each group in each test. Significant differences from baseline are indicated by ^∗^*P* < 0.05.

We next wanted to see if HIF-1*α* played a role in regulating SNAI1 expression in human pulmonary microvascular endothelial cells. Following the silencing of *HIF-1α* in female HPMECs, *SNAI1* mRNA (Figure 5(a)) and protein expression (Figure 5(b)) were decreased in female HPMECs.

#### 3.3.3. SNAI1 Expression following miR30a-5p Inhibition, HIF-1*α* Overexpression, or Both in Female HPMECs

**Figure 6:**
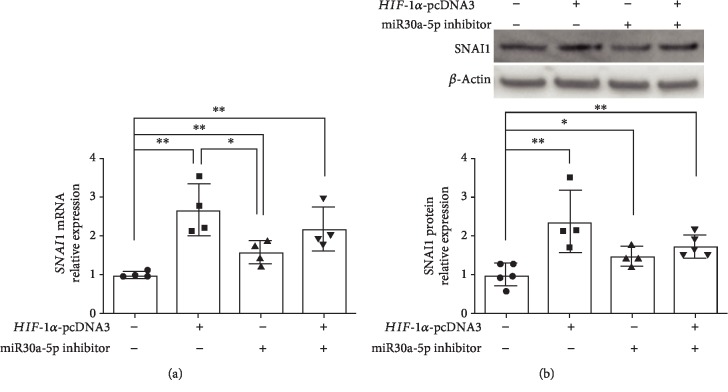
*SNAI1* expression following miR30a-5p inhibition, HIF-1*α* overexpression, or both in female HPMECs: *SNAI1* mRNA (a) and protein (b) expression in female HPMECs transfected with HIF-1*α* overexpression plasmid (*HIF-1α*-pcDNA3), miR30a-5p inhibitor, or both. Values are expressed as mean ± SD. *N* = 4 experimental replicates for each group. Significant differences between the indicated groups are shown by ^∗^*P* < 0.05 and ^∗∗^*P* < 0.01.

Since both miR30a-5p and *HIF-1α* had opposing effects on SNAI1 expression, we wanted to determine the independent and additive effects of *miR30a-5p* inhibition and *HIF-1α* overexpression in female HPMECs. *HIF-1α* overexpression and *miR30a-5p* inhibition both independently increased SNAI1 mRNA expression in female HPMECs (Figure 6(a)). *HIF-1α* overexpression had a more significant effect on SNAI1 expression compared to *miR30a-5p* inhibition. Combined *HIF-1α* overexpression and *miR30a-5p* inhibition increased SNAI1 expression compared to controls, but there was no additive effect (Figure 6(a)). SNAI1 protein was increased by *miR30a-5p* inhibition or *HIF-1α* overexpression independently and by the combined treatment with *miR30a-5p* inhibition and *HIF-1α* overexpression compared to controls. No statistically significant changes in protein levels were noted between the individual treatment groups (Figure 6(b)).

## 4. Discussion

**Figure 7:**
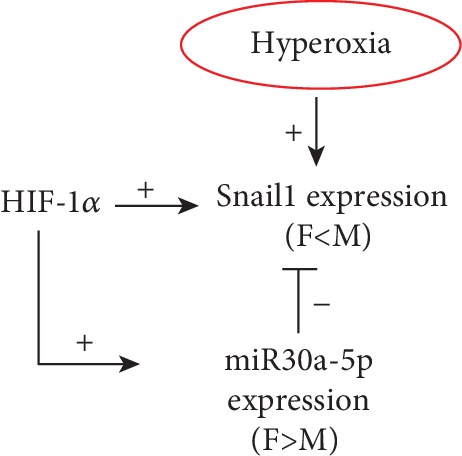
Overall schematic. Hyperoxia increases SNAI1 expression in vitro, but females show decreased expression in vivo. miR-30a decreases Snai1 expression, and females show higher expression. HIF-1*α* increases both the miR-30a and Snai1 expressions, but HIF-1*α*-mediated miR-30a expression in females keeps the Snai1 expression in check upon exposure to hyperoxia.

Sex-specific differences exist in many neonatal morbidities including bronchopulmonary dysplasia (BPD), which adversely affects a significant number of extremely premature newborns. Elucidation of mechanisms responsible for the sex-specific differences in the development of this disease is critical for the development of individualized therapies. We identified miR-30a as a potential modulator of many of the genes differentially expressed in the angiogenesis pathway [9]. Interestingly, miR-30a expression was increased in the female lung after exposure to hyperoxia. These findings were replicated in neonatal human pulmonary microvascular endothelial cells (HPMECs), with higher miR-30a expression in female HPMECs upon exposure to hyperoxia. miR-30a modulates many biological processes and has proangiogenic and antifibrotic properties. A miRNA can have many putative mRNA targets. *Snai1* (a profibrotic gene) is a miR-30a target. We show that Snai1 expression is decreased in female neonatal mice *in vivo*. On the other hand, male HPMECs show increased SNAI1 expression *in vitro* after exposure to hyperoxia. Also, we show that *Hif-1α* could modulate miR-30a expression in HPMECs. Interestingly, *Hif-1α* also increases *Snai1* expression, and the net protective effect in females could be mediated through a *Hif-1α*-mediated increase in the *miR-30a* expression which keeps the *Snai1* expression in check during exposure to hyperoxia (Figure 7).

*Snai1* (Snai Family Transcriptional Repressor 1) is a zinc finger transcriptional repressor protein and plays a central role in both epithelial to mesenchymal (EMT) [30] and endothelial to mesenchymal (Endo-MT) in many disease processes. Exposure to hyperoxia leads to a profibrotic phenotype leading to an increase in myofibroblasts [31–34]. Endo-MT is a complex biological process in which endothelial cells lose their surface expression of endothelial-specific markers (e.g., CD31 and vWF) and acquire a mesenchymal phenotype (elongated and fusiform) and express mesenchymal markers (like *α*-SMA and vimentin) [35]. The role of Endo-MT is known in lung diseases such as idiopathic pulmonary fibrosis (significant contribution to fibrosis and vascular regression) and pulmonary arterial hypertension (by causing pulmonary vascular remodeling and endothelial dysfunction) [36–38]. We have previously shown that male HPMECs show higher expression of *α*-SMA upon exposure to hyperoxia accompanied by a decrease in CD31 expression [39]. In this study, *SNAI1* mRNA expression was significantly higher in male HPMECs compared to female. Male HPMECs showed increased SNAIL1 protein upon exposure to hyperoxia, while female HPMECs did not show this change. In vivo, *Snai1* expression was decreased in females immediately after (P7) hyperoxia exposure.

Since our group had discovered sex-specific differences in *miR-30a* expression with females showing higher expression than males [9], we wanted to elucidate the possible regulation of *SNAI1* expression by *miR-30a* in female HPMECs. Other studies have shown *miR-30a* mediated downregulation of *Snai1* [10, 14, 17]. Zhang et al. reported that *miR-30a* targeted and downregulated *SNAI1* in a diabetic cataract model *in vitro*. Similar antifibrotic effects were reported in non-small cell lung cancer [40], atrial fibrillation-induced myocardial fibrosis [14], and TGF-*β*1-induced peritoneal fibrosis [10]. In female HPMECs, in the present study, we show decreased expression of *SNAI1* with *miR-30a* overexpression and increased expression with *miR-30a* inhibition. To our knowledge, this is the first study to show that *miR-30a* targets *SNAI1* in neonatal HPMECs.

Even though we had reported sex-specific differences in *miR-30a* expression [9], upstream modulation of *miR-30a* was not elucidated. *Hif-1α* is expressed in early lung development and slowly decreases expression as the lung matures, with a second transient peak at the alveolar stage of lung development around PND10 [20, 41]. Use of prolyl hydroxylase domain (PHD) inhibitors to increase HIF protein levels improved lung growth in a preterm baboon model of BPD [42] Interestingly, *Hif-1α* activity is increased, when human umbilical vein endothelial cells are returned to normoxic conditions after being exposed to hyperoxia [43]. We had previously reported that *HIF-1α* binding to its target genes is more significant in female lungs after hyperoxia exposure [22]. In contrast to male mice, females did not show a decrease in *Hif-1α* expression at PND21 after early hyperoxia exposure. Yang et al. showed that HIF-1*α* inhibition abrogated *miR-30a* upregulation in cardiomyocytes [18]. In this study, we were able to show that the silencing of *HIF-1α* in HPMECs significantly decreased *miR-30a* expression in HPMECs, thus postulating a role for *HIF-1α* in increasing *miR-30a* expression in the hyperoxia model, especially during the normoxic recovery phase after exposure to hyperoxia.

Many factors including HIF-1*α*, NFKB, STAT3, and NICD can induce Snai1 expression [30]. Xu et *a*l. showed that *HIF-1α* could induce *Snai1*, independent of *TGF-β*, and that *SNAI1* is a direct target of *HIF-1α* in human coronary endothelial cells [23]. We wanted to see if *HIF-1α* modulated SNAI1 expression in HPMECs. *HIF-1α* knockdown in HPMECs decreased *SNAI1* mRNA and protein expression. Similar results were reported by Zhu et al. in pancreatic cancer cells [44]. Hypoxia-induced Endo-MT in a model of radiation-induced pulmonary fibrosis was mediated through *HIF-1α* [45]*. HIF-1α* may be thus inducing both profibrotic (*SNAI1*) and antifibrotic (*miR-30a*) pathways in HPMECs. The balance of expression between the two mediators may eventually predispose or protect the lung from pathologic fibrosis. In our study, *HIF-1α* overexpression and *miR30a-5p* inhibition either separately or in combination increased *SNAI1* expression, but the combined treatment did not produce an additive effect in *SNAI1* expression.

We recognize the following limitations in this study. Our study only evaluated two time points *in vivo* after hyperoxia exposure during the saccular stage of lung development in mice. Later time points may have sustained differences in *Snai1* expression, or these differences may be lost. Also, we used high oxygen concentration (0.95 FiO_2_) for our hyperoxia exposure protocol based on the reproducible BPD-like phenotype produced in mice. Even though this limits hyperoxia exposure to the saccular stage of lung development, which equates to 26-36 weeks in human neonates, this oxygen concentration is much higher than what is used in the human NICU. Also, these results may be different from other models, where hyperoxia exposure is continued for longer durations [2]. We are also aware that findings in the murine model may be strain-specific [46]. Our *in vivo* data is from whole lung tissue and does not show endothelial-specific expression. Our future studies will seek to validate the cell-specific expression of *Snai1* in primary mouse endothelial cells and BPD and non-BPD patient samples. In conclusion, we show that the intersection between *HIF-1α*, *miR-30a*, and *Snai1* may play a role in driving sex-specific differences in after neonatal hyperoxic lung injury.

## Data Availability

The data used to support the findings of this study are available from the corresponding author upon request.

## Supplementary Materials

Supplementary Materials Supplementary Figure 1: Snai1 expression in vivo and in vitro: Snai1 mRNA (A–C) and protein (D–F) expression in neonatal male and female mice exposed to hyperoxia (P1-5, 0.95 FiO2) during the saccular stage of lung development at P7 (A, D) and P21 (B, E) and in male and female neonatal human pulmonary microvascular endothelial cells (HPMECs) (C, F). Significant differences between the indicated groups is shown by ^∗^*P* < 0.05.
