# Supplementary material for: Role of HIF-1α-miR30a-Snai1 Axis in Neonatal Hyperoxic Lung Injury
Source: Oxid Med Cell Longev. 2019 Oct 22;2019:8327486. doi: 10.1155/2019/8327486 (PMC6854945; doi:10.1155/2019/8327486)

Supplemental Figure 1: Snai1 expression *in vivo* and *in vitro: Snai1* mRNA (normalized to Beta-2 microglobulin) (A-C) and protein (D-F) expression (normalized to vinculin expression) in neonatal male and female mice exposed to hyperoxia(P 1-5; 95%FiO2) during the saccular stage of lung development at P7 (A,D) and P21 (B,E) and in male and female neonatal human pulmonary microvascula endothelial cells (HPMECs); (C,F). Significant differences between the indicated groups is shown by *P<0.05.

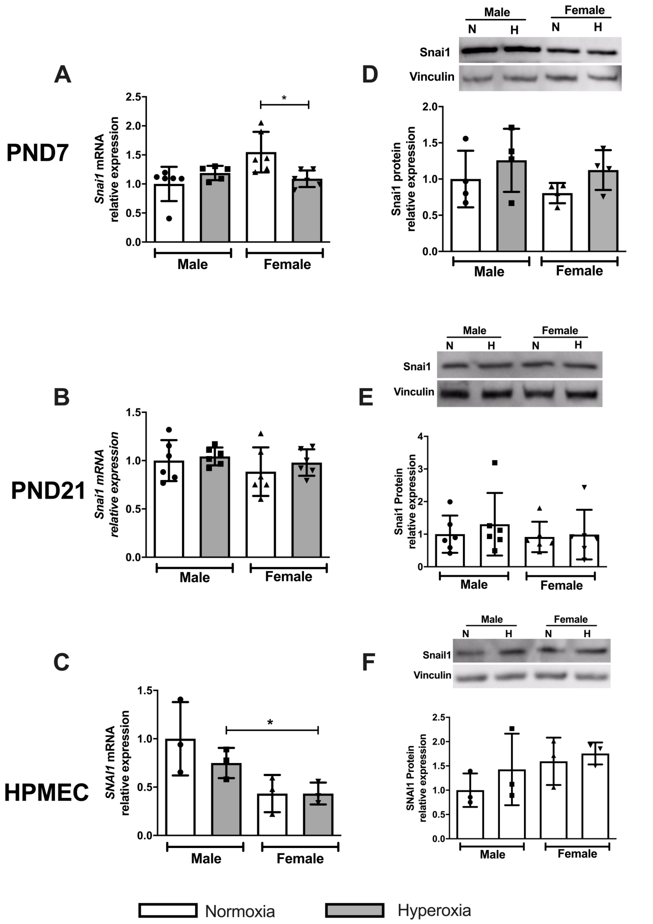

Supplement: Supplementary Materials — Supplementary Figure 1: Snai1 expression in vivo and in vitro: Snai1 mRNA (A–C) and protein (D–F) expression in neonatal male and female mice exposed to hyperoxia (P1-5, 0.95 FiO2) during the saccular stage of lung development at P7 (A, D) and P21 (B, E) and in male and female neonatal human pulmonary microvascular endothelial cells (HPMECs) (C, F). Significant differences between the indicated groups is shown by ∗P < 0.05. [file 8327486.f1.pdf]
